# Place and Child Health: The Interaction of Population Density and Sanitation in Developing Countries

**DOI:** 10.1007/s13524-016-0538-y

**Published:** 2017-01-09

**Authors:** Payal Hathi, Sabrina Haque, Lovey Pant, Diane Coffey, Dean Spears

**Affiliations:** 1 r.i.c.e., a research institute for compassionate economics, New Delhi, India; 2 World Bank Water and Sanitation Program, Washington, DC USA; 3 Department of Sociology & Population Research Center, University of Texas, Austin, TX USA; 4 Indian Statistical Institute, Delhi, India; 5 Department of Economics, University of Texas, Austin, TX USA

**Keywords:** Sanitation, Population density, Infant mortality, Child height, South Asia

## Abstract

A long literature in demography has debated the importance of place for health, especially children’s health. In this study, we assess whether the importance of dense settlement for infant mortality and child height is moderated by exposure to local sanitation behavior. Is open defecation (i.e., without a toilet or latrine) worse for infant mortality and child height where population density is greater? Is poor sanitation is an important mechanism by which population density influences child health outcomes? We present two complementary analyses using newly assembled data sets, which represent two points in a trade-off between external and internal validity. First, we concentrate on external validity by studying infant mortality and child height in a large, international child-level data set of 172 Demographic and Health Surveys, matched to census population density data for 1,800 subnational regions. Second, we concentrate on internal validity by studying child height in Bangladeshi districts, using a new data set constructed with GIS techniques that allows us to control for fixed effects at a high level of geographic resolution. We find a statistically robust and quantitatively comparable interaction between sanitation and population density with both approaches: open defecation externalities are more important for child health outcomes where people live more closely together.

**Electronic supplementary material:**

The online version of this article (doi:10.1007/s13524-016-0538-y) contains supplementary material, which is available to authorized users.

## Introduction

A long literature in demography has explored the importance of place for health (Entwisle 2007). In many cases, these studies have been characterized as a debate over the health consequences of living in urban settings versus rural settings (Dye 2008; Sastry 1997; Woods 2004). Although many demographers UNICEF study the effects of urban residence on health in developed countries have found a strong urban advantage (Eberhardt et al. 2001; Hartley 2004), discussions of urban health have often historically begun with the history of poor sanitation and high infectious disease burdens that plagued the cities of now-rich countries while they were developing (Cutler and Miller 2005; Preston 1975).

In modern developing countries, there is active debate about what defines “urbanness” (Dorélien et al. 2013; Hugo et al. 2003) and when and why urban advantages in infant and child health exist (Fink et al. 2014; Günther and Harttgen 2012; Jankowska et al. 2013; Montgomery and Hewett 2005; Smith et al. 2005; Van de Poel et al. 2007). Bocquier et al. (2011) pointed out that urban advantages depend on the services and economic opportunities that a city provides, whereas Sastry (1996) noted that the effects of community-level variables on child health often depend on context—that is, that when exploring the effects of place on health, interactions are often important.

In developing countries, dense settlement often implies a number of health advantages for children. For example, dense settlement is correlated with more wealth (which buys better housing and food) and with more schooling (which leads to better-educated mothers). Additionally, people in densely populated areas are more likely to have access to health services that matter for child survival and development, such as trained doctors, maternal care, and medicines (Magadi et al. 2003; Matthews et al. 2010).

However, scholars have also hypothesized that one important reason why place matters for health in developing countries today—and why it mattered in developed countries historically—is variation in sanitation and the disease environment (McGuire and Coelho 2011; Mosley and Chen 1984; Preston and Haines 1991). Recent research in economics, epidemiology, and public health has suggested that *open defecation*—the practice of defecating in the open without using a toilet or latrine—is an important cause of infant mortality and child stunting in both rural and urban areas of developing countries (Cameron et al. 2013; Fink et al. 2011; Humphrey 2009; Spears 2013).

In this article, we assess whether the importance of dense settlement for infant mortality and child height is moderated by exposure to local or community-level sanitation behavior. We also ask whether sanitation interacts with population density to produce these child health outcomes. Such an interaction would be consistent with facts and theories in the literature. If open defecation reduces human capital by releasing germs into children’s environments, it is plausible that the consequences of open defecation would be worse where people live more closely together and are more likely to encounter their neighbor’s germs.

Documenting and measuring the magnitude of the interaction between open defecation and population density is important for several reasons. First, it moves beyond dichotomous rural and urban distinctions and clarifies the circumstances under which population density is positively associated with health, and the circumstances under which poor sanitation is particularly harmful. Second, it contributes to understanding the importance of externalities or “spillover effects” of sanitation: one household’s toilet use or open defecation has consequences for neighboring households’ children. Such externalities are recognized in public economics as a central rationale for policy action. Finally, documenting and measuring such an interaction could guide policy decisions. Open defecation is increasingly concentrated in South Asia, a region where even rural areas are very densely populated, making the region an important area of focus for sanitation policy.

We present two complementary analyses: the first establishes the broad importance of the interaction between sanitation behavior and population density for predicting infant mortality and child height in developing countries, and the second provides evidence to support the internal validity of this interaction. For the first analysis, we construct a new international data set from 172 Demographic and Health Surveys (hereafter, DHS) collected in 69 developing countries between 1990 and 2012. Child-level health data are matched with estimates of community open-defecation rates and census population density data for 1,800 subnational regions. For the second analysis, we use geographic information system (GIS) codes to create a new data set of children in Bangladesh that allows us to identify the effect of the interaction of population density with local sanitation on child height. These new data allow our measure of population density to be more precise than is possible in the international data set, and they allow us to control for higher-resolution geographic fixed effects.

We motivate the international analysis by confirming the results of prior studies showing that urban children in developing countries are less likely to die in the first year of life than rural children. Using the data set of 172 DHS, we find that part of this difference is explained by the fact that rural children are exposed to more open defecation, on average, than urban children. However, a positive interaction of urban place with local open defecation suggests that the urban survival advantage is less pronounced where open defecation is high. Further controlling for the interaction of population density and local sanitation clarifies that higher average population density in urban areas is the mechanism through which urban residence likely moderates the effect of sanitation on infant mortality.

We then focus directly on the population density–sanitation interaction and show that it is robust to a variety of respecifications. We also perform falsification tests to show that other variables related to socioeconomic status (SES) do not similarly interact with population density to predict infant mortality in these data. Finally, we plot the shape of the interaction between local open defecation and population density and find that it is steeper at higher population densities.

The second analysis seeks to further test the internal validity of the interaction between sanitation and population density in predicting child height. We use GIS codes to match children in the Bangladesh DHS to the population density for their area of residence using highly disaggregated census data. This approach allows us to construct an interaction of population density and local sanitation that provides a more precise measure of exposure to density of open defecation than we are able to use in the international data set. We then regress child height on these more precise measures of exposure to density of open defecation using district and survey round fixed effects. As in Sastry and Hussey (2003), we use geographic fixed effects because they control for time-invariant properties of place at the level of the fixed effect—in this case, the district.^1^ The magnitude of the interaction that we identify in the Bangladesh data set is quantitatively similar to what is predicted for Bangladesh by a semiparametric model fit to the international data.

This article proceeds in three sections. We first summarize evidence from the literature about why poor sanitation would be expected to have a larger effect on infant mortality and child height where population density is higher. Then we describe the analysis and present results from the international data set. Next, we describe the analysis and present results from the Bangladesh data set, and then discuss the findings. We point out that although taken at face value, our results might seem to recommend concentrating policy efforts on improving sanitation in urban areas, the distributions of sanitation coverage and population density in the world today show that many of the places where open defecation is most densely practiced are actually classified as rural. Indeed, our findings, combined with these empirical distributions, highlight the threats to child health posed by the enduring density of open defecation, particularly in rural South Asia.

## Background: Population Density, Sanitation, and Disease Externalities

On average, rural places have lower population density than urban places but also have more open defecation than urban places and lower-quality sanitation. Although developing countries are making progress in improving sanitation, more than 1 billion people still defecate in the open, without using a toilet or latrine (UNICEF and WHO 2012). Increasingly, open defecation is concentrated in rural areas, but it is also becoming increasingly concentrated in countries with high rural population densities, such as Indonesia, Pakistan, and especially India (Coffey et al. 2014), where the 2011 census found that 90 % of households without a toilet or latrine live in rural areas.

Open defecation is a practice with strong negative health externalities: it spreads infectious diseases—such as diarrhea, polio, cholera—and parasites. Greater population density could exacerbate these negative externalities by providing more opportunities for disease transmission. Despite several examples of population density–health interactions in present-day developing countries in the literature^2^ and evidence from present-day developed countries,^3^ discussion of the evidence that population density can intensify an epidemiological externality often begins with the history of urbanization in now-rich countries.

Much has been written about the lethal combination of population density and poor sanitation in nineteenth century London. To illustrate an exemplary use of observational statistics, Freedman (1991) recounted John Snow’s investigation of the 1853–1854 cholera epidemic. By tracing deaths to the supply of their households’ water, Snow demonstrated the nature of the epidemic and is widely credited for establishing the infectious mechanism of the disease.

A large medical and epidemiological literature has documented that poor sanitation continues to cause death and disease, particularly among children in developing countries. Ingestion of fecal pathogens as a result of living near poor sanitation is well known to cause diarrhea (Esrey et al. 1991). Checkley et al. (2008) used detailed, high-frequency longitudinal data from five countries to demonstrate effects of childhood diarrhea on subsequent height. Humphrey (2009) posited that chronic but subclinical “environmental enteropathy,” caused by ingestion of fecal pathogens, may also lead to slowed growth. Lin et al. (2013) found associations among fecal environmental contamination, enteropathy, and child height in Bangladesh. Poor sanitation can also spread parasitic infections, which are rarely fatal by themselves but contribute to poor health and poor physical growth (Haque 2007).^4^ Several studies in economics have also identified important effects of sanitation-related diseases on anemia and early-life mortality (e.g., Coffey et al. Forthcoming; Cutler and Miller 2005; Galiani et al. 2005; Watson 2006) as well as effects on subsequent human capital accumulation (e.g., Baird et al. 2016; Bleakley 2007; Hammer and Spears 2016; Spears and Lamba 2016).

Recent econometric studies have suggested an interaction between sanitation and population density in predicting health and human capital outcomes across developing countries. As motivation for a study that seeks to explain differences in child height between India and sub-Saharan Africa, Spears (2013) observed that heterogeneity in open-defecation density across developing countries accounts for a large fraction of international differences in average child height. However, Spears (2013) did not focus on the internal validity of the sanitation–population density interaction. The following analyses are the first to use micro-level data from all available DHS and disaggregated fixed effects to quantify and verify the robustness of this interaction.

## Population Density, Sanitation, and Child Health in Developing Countries: Evidence From 172 DHS

In these analyses, we use a data set of 172 DHS collected between 1990 and 2012 in 69 developing countries to assess whether the importance of dense settlement for infant mortality and child height in developing countries is moderated by exposure to local sanitation behavior.

As motivation, we begin with a description of how urban place, sanitation, and population density predict infant mortality. We find that the urban infant survival advantage is importantly diminished after we control for local sanitation, population density, and their interaction. We then focus directly on the interaction of population density with local open defecation in predicting infant mortality and child height. Although this multicountry analysis is not intended to precisely identify a causal effect, we demonstrate that the effect of population density on the sanitation-health gradient is quantitatively robust to model respecifications, including the introduction of a range of fixed effects and controls, suggesting that the interaction we document is unlikely to reflect omitted variables. To provide additional evidence that this relationship is not due to omitted variables, we conduct falsification tests that demonstrate that other measures of SES do not similarly interact with population density to predict infant mortality. Finally, we model the shape of the dependence of the sanitation-mortality gradient and the sanitation-height gradient on population density.

### Data and Summary Statistics

These analyses combine data from two sources: (1) population density from census or other aggregate demographic data; and (2) sanitation, health, and other covariate data from DHS collected between 1990 and 2012. DHS are internationally comparable, nationally representative surveys collected in poor and middle-income countries.^5^ We append all available DHS to make a large data set in which each observation is an individual child. We merge to the child-level data a new data set on population density at the level of DHS subnational regions (hereafter, regions). For each of the more than 1,800 regions, we manually matched the region to publicly available, published demographic data for the closest available year to the year of the survey. Table S1 in Online Resource 1 lists all the countries and years in the international sample as well as the source of the region level data on population density.

#### Independent Variable of Interest

Our independent variable of interest is the interaction of the log of population density at the region level with local prevalence of open defecation near a child. We estimate local prevalence of open defecation near a child by estimating the fraction of the households in a child’s primary sampling unit (PSU)^6^ that defecate in the open rather than using a toilet or latrine. We do this by computing the fraction of households in each PSU in the sample that report open defecation.^7^ This is a local (or community-level) measure of exposure to open defecation, and not merely a property of the child’s own household (Montgomery and Hewett 2005). To isolate and emphasize the negative externality of neighbors’ open defecation, we also control for whether a child’s own household defecates in the open in all the regressions that we present.

#### Dependent Variables

Our dependent variables are infant mortality and height-for-age. Infant mortality is a child-level indicator, which we define for all live births that occurred at least one year before the date of the survey and no more than five years before the date of the survey.^8^ Infant mortality is coded as 0 if the child survived her first year of life, and as 1,000 if the child died within the first year. This scaling of the indicator by 1,000 makes our infant mortality estimates consistent with published population-level infant mortality rate (IMR) statistics. The second dependent variable is a child’s height-for-age *z* score.^9^ A height-for-age *z* score scales a child’s height relative to a healthy population of that child’s age and sex. We use the 2006 WHO international reference population of healthy children.

#### Summary Statistics

Table 1 presents summary statistics about the international data set. Panel A shows summary statistics for the dependent, independent, and select control variables in the sample as a whole; panels B and C show summary statistics for children living in below-median and above-median open-defecation PSUs, respectively.

**Table 1:** Summary statistics, international sample

	Mean	25th Percentile		Median	75th Percentile
Panel A: Full Sample					
Infant mortality rate (IMR)	62.24				
Height-for-age	–1.49	–2.59		–1.53	–0.47
Local open defecation	0.35	0.00		0.14	0.72
Household open defecation	0.35	0		0	1
Population density per km^2^	443	31		81	239
ln(Density)	4.48	3.43		4.39	5.47
GDP per capita (USD)	1,079	324		525	1,249
Local piped water	0.28	0		0	0.57
Local electrification	0.41	0		0.22	0.92
Urban	0.33	0		0	1
Mother ever attended school	0.61	0		1	1
Mother’s age at first birth	19	17		91	21
Mother’s height (cm)	130	126		130	134
*n* (IMR: live births)	1,112,465				
*n* (height: children under 5)	858,514				
Panel B: Below-Median Local Open Defecation					
Infant mortality rate	50.98				
Height-for-age	–1.31		–2.32	–1.28	–0.28
Local open defecation	0.03		0	0	0.06
Household open defecation	0.03		0	0	0
Population density per km^2^	677		39	91	308
ln(Density)	4.71	3.66		4.51	5.73
GDP per capita (USD)	1,379		360	771	1,718
Local piped water	0.59		0.04	0.81	1.00
Local electrification	0.41		0.00	0.25	0.88
Urban	0.53		0	1	1
Mother ever attended school	0.76		1	1	1
Mother’s age at first birth	20		17	19	22
Mother’s height (cm)	130		127	130	134
Panel C: Above-Median Local Open Defecation					
Infant mortality rate	73.39				
Height-for-age	–1.85	–2.95		–1.87	–0.78
Local open defecation	0.67	0.40		0.72	0.95
Household open defecation	0.66	0		1	1
Population density per km^2^	211	26		72	203
ln(Density)	4.25	3.26		4.28	5.31
GDP per capita (USD)	780	324		441	783
Local piped water	0.24	0		0	0.46
Local electrification	0.14	0		0	0.12
Urban	0.13	0		0	0
Mother ever attended school	0.46	0		0	1
Mother’s age at first birth	19	17		18	21
Mother’s height (cm)	130	125		130	134

*Notes:* Observations are individual children born alive in the 10 years before the survey. Children are included in the summary statistics sample if they are in either the IMR or the height sample.

More than 6 % of children in the data died before their first birthday. The average child in our data is notably shorter than children in the healthy reference population. Children in above-median open-defecation PSUs are approximately 0.5 standard deviations, on average, shorter than children in below-median open-defecation PSUs. Approximately one-third of the average child’s neighbors defecate in the open.

Population density varies widely in our sample, with an interquartile range from 31 to 239 people per square kilometer. Children living in above-median open-defecation PSUs live in less population–dense regions, on average, than those in below-median open-defecation PSUs. Fig. S2 of Online Resource 1 plots a kernel density estimate of the distribution of population density among children in our international sample. Throughout our analysis, we transform population density to a log scale. A normal distribution with the same mean and standard deviation is included for comparison; population density appears to match a lognormal distribution.

Although it is not used in the regressions (because it would be a country-year fixed effect, which we use as a control), we include GDP per capita from the Penn World Tables in Table 1 for illustration. The median child in this data set is poor: she is growing up in a country-year with a GDP per capita per day of $1.44. Finally, we also present summary statistics for some of the variables that we use in falsification tests and for some of the mother-level controls used in the regressions. More than one-quarter (28 %) of the children’s neighbors have piped water, and 41 % of them have electricity. PSU average piped-water access and electrification are far lower for children living in PSUs with above-median open-defecation rates. More than one-half (61 %) of children had a mother who ever attended school, and the median child’s mother was 19 years old when she first gave birth.

### Motivation: Urban Place, Sanitation, and Infant Mortality

The literature reviewed thus far suggests that, on average, dense settlement in developing countries confers an infant health advantage. However, the literature also suggests that this advantage will be less pronounced where sanitation is poor. In this section, we motivate the analyses that follow by using the international data set to present results from regressions of the following form:

1 $$ {mortality}_{ip}={\upbeta}_1{place}_p+{\upbeta}_2 open\ {defecation}_p+\left({\upbeta}_3{place}_p\times open\ {defecation}_p\right)+{\upalpha}_c+{\upvarepsilon}_{ip}, $$

where *mortality* for child *i* living in place *p* is scaled for infant deaths per 1,000; *open defecation* is open defecation in the child’s local area (PSU); α_*c*_ is a country fixed effect; and *place* will be implemented either as a dummy variable for urban residence (as defined by the DHS),^10^ as population density of the child’s subnational region, or with both in the same regression. All the variables are demeaned to facilitate comparability of coefficients across columns.

Table 2 presents descriptive regression results that build on the result of Eq. (1). We begin by estimating the within-country urban infant survival advantage in our data set. Column 1 shows that averaging over the combined data set, children in urban places (as defined by the DHS) are 16 per 1,000 more likely to survive their first year of life than children in rural places. Part of this apparently large urban advantage reflects the better sanitation environment in urban areas than rural areas. Column 2 adds local sanitation and shows that controlling for the better sanitation environment in cities diminishes the urban coefficient. However, this model assumes that open defecation has the same association with infant mortality in both urban and rural areas. Column 3 includes the interaction of urban and local sanitation, and finds that the coefficient on urban declines in absolute magnitude by almost two-thirds relative to the magnitude of its coefficient in column 1. Open defecation and urban residence interact: open defecation is more steeply associated with mortality in urban rather than rural places. The average urban child is only 2.4 per 1,000 less likely to die in infancy in places where everyone defecates in the open, compared with 8.0 per 1,000—or more than triple the advantage—in places where nobody defecates in the open.^11^

**Table 2:** Urban residence, population density, sanitation, and mortality: International sample

	Infant Mortality Deaths per 1,000					
	(1)	(2)	(3)	(4)	(5)	(6)
Urban	–16.06***	–7.050***	–6.047***			–5.751**
	(1.502)	(1.614)	(1.753)			(1.760)
Local Open Defecation		27.22***	28.28***		32.38***	28.71***
		(3.945)	(4.119)		(2.924)	(3.742)
Urban × Local Open Defecation			5.592^†^			4.472
			(3.256)			(3.288)
ln(Density)				–2.121***	–0.331	0.0357
				(0.578)	(0.645)	(0.626)
ln(Density) × Local Open Defecation					3.321*	2.929*
					(1.381)	(1.366)
*n* (live births)	1,112,465	1,112,465	1,112,465	1,112,465	1,112,465	1,112,465

*Notes:* Standard errors are clustered by 172 DHS. All regressions include a country fixed effect. Interacted variables are demeaned to preserve interpretation across columns.

^†^ *p* < .10; *p < .05; **p < .01; ***p < .001 (two-sided tests)

Why does urban place interact with sanitation to predict infant mortality? We propose that the population density of urban places leads to greater disease externalities. Therefore, columns 4 and 5 replicate the results in columns 1 and 3, this time replacing *urban* with population density. On average, higher population density places have slightly lower infant mortality than lower population–density places. We find that open defecation is more steeply associated with mortality in more densely populated places. Population density is not itself associated with either a mortality advantage or a mortality disadvantage at the average level of open defecation.

Finally, the regression results in column 6 present a “horse race” demonstrating that increased population density is indeed the reason why urban place interacts with sanitation to predict infant mortality. We include both the interaction of population density and local sanitation, as well as urban residence and local sanitation. After the interaction of population density and open defecation is accounted for, there is no longer an apparent interaction between urban place and sanitation. Population density per se appears neither associated with greater nor lesser mortality, and the urban advantage documented in column 6 is only one-third as much as appeared to be the case in column 1.

Table 2 suggests that in developing countries, an interaction between sanitation and population density importantly moderates the relationship between place and early-life health and mortality. The analyses that follow sharpen our understanding of this interaction and investigate its external and internal validity.

### The Interaction of Sanitation and Population Density in 172 DHS

We have seen that the relationship between urban place and health depends importantly on population density and on open defecation. In this section, we focus directly on establishing an interaction between population density and sanitation, and then assess the robustness of the estimate.

#### Empirical Strategy

For each dependent variable, we regress health on a linear interaction of local sanitation and population density, controlling for household sanitation and one of three levels of fixed effects α:

- *country*: For example, a fixed effect for India, pooling over the 1992, 1998, and 2005 DHS
- *survey*: A partition of *country*: for example, a fixed effect for India in each surveyed year
- *region*: A partition of *survey* into the subnational region level at which population density is matched—for example, the Indian state of Bihar in 2005

Note that adding fixed effects means that our identification is derived from heterogeneity within these regions. Depending on the question we seek to answer, this may be *over*controlling. For example, in the case of the region fixed effects, the difference in population density between regions within the same country may be of policy relevance.

Our regression specification is as follows:

2 $$ \begin{array}{l}{health}_{ipsc}={\upbeta}_1 local\ {OD}_{ipsc}\times \ln \left({density}_{psc}\right)+{\upbeta}_2 \ln \left({density}_{psc}\right)+{\upbeta}_3 local\ {OD}_{ipsc}\\ {}+{\upbeta}_4 household\ {OD}_{ipsc}+{\mathbf{X}}_{ipsc}\uptheta +{\upalpha}_{psc}+{\upvarepsilon}_{ipsc},\end{array} $$

where *i* indexes individual children, *p* is the region for which population density is matched, *s* indicates a DHS, and *c* is a country. **X** is an extensive set of controls which we use throughout the analysis of the international data set. It includes indicators for the child’s household owning the six common DHS assets (electricity, radio, television, motorcycle, car, and refrigerator); indicators for sex, birth calendar month, and multiple births; year of birth entered linearly; indicators for first, second, or third birth order; an indicator for whether the child’s mother attended school; and the mother’s age at first birth entered linearly.^12^ We also control for whether the child’s own household defecates in the open. When child height is the dependent variable, we always add a vector of 120 age (in months) by sex indicators. Standard errors are conservatively clustered at the level of 172 DHS (thus, India’s entire 2005 DHS is one cluster), except in specifications with country fixed effects, where standard errors are even more conservatively clustered at the country level.^13^

#### Results

Table 3 reports estimates of Eq. (2), for infant mortality in panel A and for height-for-age in panel B. It reports results using several combinations of fixed effects and controls. The result is quantitatively robust as the estimates remain in a stable range: a one log-unit increase in population density increases the change in infant mortality associated with moving from no neighbors defecating in the open to all neighbors defecating in the open by about 2 deaths per 1,000 live births, and increases the corresponding decline in height-for-age by about 0.04 of a height-for-age standard deviation.^14^

**Table 3:** Local open defecation robustly linearly interacts with population density: International sample

	Fixed Effects					
	Country	Country	Survey	Survey	Region	Region
	(1)	(2)	(3)	(4)	(5)	(6)
Panel A: Infant Mortality Is the Dependent Variable						
Local open defecation *×* ln(Density)	3.273*	2.271*	3.523**	2.772*	2.266*	1.581
	(1.390)	(1.049)	(1.178)	(1.077)	(1.060)	(1.071)
Local open defecation	26.27***	12.61***	22.99***	11.71***	18.80***	8.715***
	(2.339)	(2.244)	(1.978)	(2.186)	(1.794)	(2.166)
ln(Density)	–0.330	0.518	–0.316	0.390		
	(0.646)	(0.519)	(0.518)	(0.495)		
Household open defecation	6.246***	3.102**	6.141***	3.455***	6.276***	3.808***
	(1.711)	(1.049)	(1.309)	(1.015)	(1.278)	(1.021)
Urban		–1.709		–2.252		–2.222^†^
		(2.051)		(1.446)		(1.152)
Extended controls		✓		✓		✓
*N* (live births)	1,109,116	942,350	1,109,116	942,350	1,109,116	942,350
Panel B: Child Height-for-Age Is the Dependent Variable						
Local open defecation *×* ln(Density)	–0.0744*	–0.0445	–0.0677**	–0.0396*	–0.0394**	–0.0229^†^
	(0.0335)	(0.0275)	(0.0218)	(0.0192)	(0.0146)	(0.0116)
Local open defecation	–0.493***	–0.115*	–0.457***	–0.102**	–0.437***	–0.114***
	(0.0465)	(0.0490)	(0.0325)	(0.0329)	(0.0236)	(0.0208)
ln(Density)	0.0259^†^	–0.00212	0.0257**	–0.00168		
	(0.0150)	(0.0133)	(0.00957)	(0.00916)		
Household open defecation	–0.183***	–0.0676***	–0.183***	–0.0718***	–0.185***	–0.0835***
	(0.0241)	(0.00840)	(0.0143)	(0.00664)	(0.0140)	(0.00657)
Urban		0.135***		0.136***		0.122***
		(0.0360)		(0.0242)		(0.0191)
Extended Controls		✓		✓		✓
Age (in months) *×* Sex	✓	✓	✓	✓	✓	✓
*n* (children under 5)	856,165	701,573	856,165	701,573	856,165	701,573

*Notes:* Standard errors are clustered by country in columns 1 and 2 and by DHS in columns 3–6. Extended controls include six indicators for the child’s household owning the six common DHS assets (electricity, radio, television, motorcycle, car, and refrigerator); indicators for sex, birth calendar month, and multiple births; year of birth, entered linearly; indicators for first, second, or third birth order; an indicator for whether the child’s mother attended school; and the mother’s age at first birth, entered linearly.

^†^ *p <* .10; *p < .05; **p < .01; ***p < .001 (two-tailed tests)

Results are similar if we use fixed effects for countries (64 for height and 69 for infant mortality), or if we instead use more than 1,800 disaggregated fixed effects by region within each survey year, with or without a long vector of controls. Indeed, the regional fixed effects may represent overcontrolling if part of what is important for child health in differences across region-years is differences in the density of open defecation across space and time. Two of the 12 coefficient estimates are not statistically significantly different from 0; we include them for completeness and note that their coefficients are of important magnitude and not statistically distinguishable from the other coefficient estimates. Moreover, this lack of statistical significance occurs only because we have conservatively clustered standard errors at country or country-year levels: if standard errors were clustered by subnational region or survey PSU (as is common in use of DHS), then both coefficients would be highly statistically significant in our very large data set.

### Falsification: Measures of SES Do Not Interact to Predict Infant Mortality

In this section, we conduct falsification tests: we interact open defecation with other “placebo” measures of community SES. If the interaction documented in Table 3 merely reflects some unobserved spurious correlation between population density and health rather than an effect of population density on the consequences of open defecation, then we would expect many other measures of community SES to similarly apparently interact with population density.^15^

**Fig. 1:**
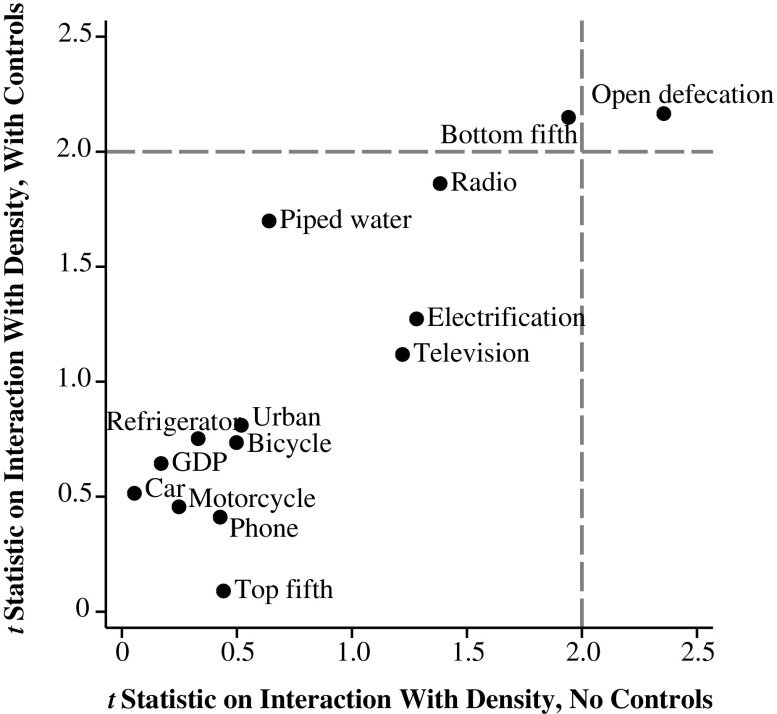
Among community-level SES measures, only open defecation interacts with population density to predict infant mortality; international sample

Figure 1 plots *t* statistics on ${\widehat{\upbeta}}_3$ from estimates of regression Eq. (3) with various community-level SES variables substituted in place of sanitation, with and without a vector of controls **X**, including the household’s own open defecation, as described earlier. Regressions take the following form:

3 $$ \begin{array}{l}{mortality}_{ipsc}={\upbeta}_1{SES}_{ipsc}+{\upbeta}_2 \ln \left({density}_{psc}\right)+\left({\upbeta}_3{SES}_{ipsc}\times \ln \left({density}_{psc}\right)\right)\\ {}+{\upbeta}_4 household\ {OD}_{ipsc}+{\mathbf{X}}_{ipsc}\uptheta +{\upalpha}_c+{\upvarepsilon}_{ipsc}.\end{array} $$

In all cases, the SES variables are community (survey PSU) averages, computed from the household recode, as in our estimated local open-defecation variable. For example, *open defecation* is the fraction of households in the PSU that defecate in the open, *radio* is the fraction of households in the PSU with a radio, and *bottom fifth* is the fraction of the PSU that the DHS asset index sorts into the bottom fifth of their survey round. The one exception is GDP, which is a country-year–level variable.

The dotted lines in Fig. 1 indicate the threshold for statistical significance. The figure shows that only local open defecation robustly statistically significantly interacts with population density to predict infant mortality, with and without controls.^16^ This specificity of the sanitation-density interaction increases our confidence that the result is indeed due to a greater effect of sanitation on height where population density is greater.

### Extension: The Shape of the Sanitation-Population Density Interaction

For tractability, the regressions in the earlier section Motivation: Urban Place, Sanitation, and Infant Mortality assumed a linear association between population density and the sanitation-health gradient: each log-unit increase in population density was assumed to be associated with the same steepening of the relationship between sanitation and health. However, with such a large data set, we can model this relationship more flexibly to show the shape of the sanitation–population density interaction.

In this section, we allow the interaction between population density and the health-sanitation gradient to be a fifth-order polynomial. We use an odd-ordered polynomial to capture flexibility in the increasing relationship between population density and the sanitation–infant mortality gradient. We use a fifth-order polynomial because of the statistical significance of these terms (*F* = 5.6; *p* < .01) and the failure of the extra sixth and seventh terms to be jointly significant additions to the model (*F* = 0.4; *p* = .79).

For both infant mortality and height-for-age, we estimate the following:

4 $$ \begin{array}{l}{health}_{ipsc}={\upbeta}_1 local\ {OD}_{ipsc}+{\displaystyle {\sum}_{j=1}^5{\upbeta}_{2,j}} \ln {\left({density}_{psc}\right)}^j+\left({\displaystyle {\sum}_{j=1}^5{\upbeta}_{3,j}}\  local\ {OD}_{ipsc}\times \ln {\left({density}_{psc}\right)}^j\right)\\ {}+\kern0.5em {\upbeta}_4 household\ {OD}_{ipsc}+{\mathbf{X}}_{ipsc}\uptheta +{\upalpha}_{psc}+{\upvarepsilon}_{ipsc}.\end{array} $$

As before, we are estimating health outcomes for child *i*, in region *p*, in DHS *s*, and in country *c*. As described earlier, we introduce fixed effects α at country, survey, and region levels in stages. We also include the same vector of extended controls, **X**, as well as the household’s own open defecation, as described earlier.

This functional form implies that the change in health associated with a change from 0 % to 100 % local open defecation is as follows:

5 $$ \frac{\partial \widehat{health}}{\partial local\ OD}={\widehat{\upbeta}}_1+{\displaystyle {\sum}_{j=1}^5{\widehat{\upbeta}}_{3,j}}\  \ln {\left({density}_{psc}\right)}^i. $$

**Fig. 2:**
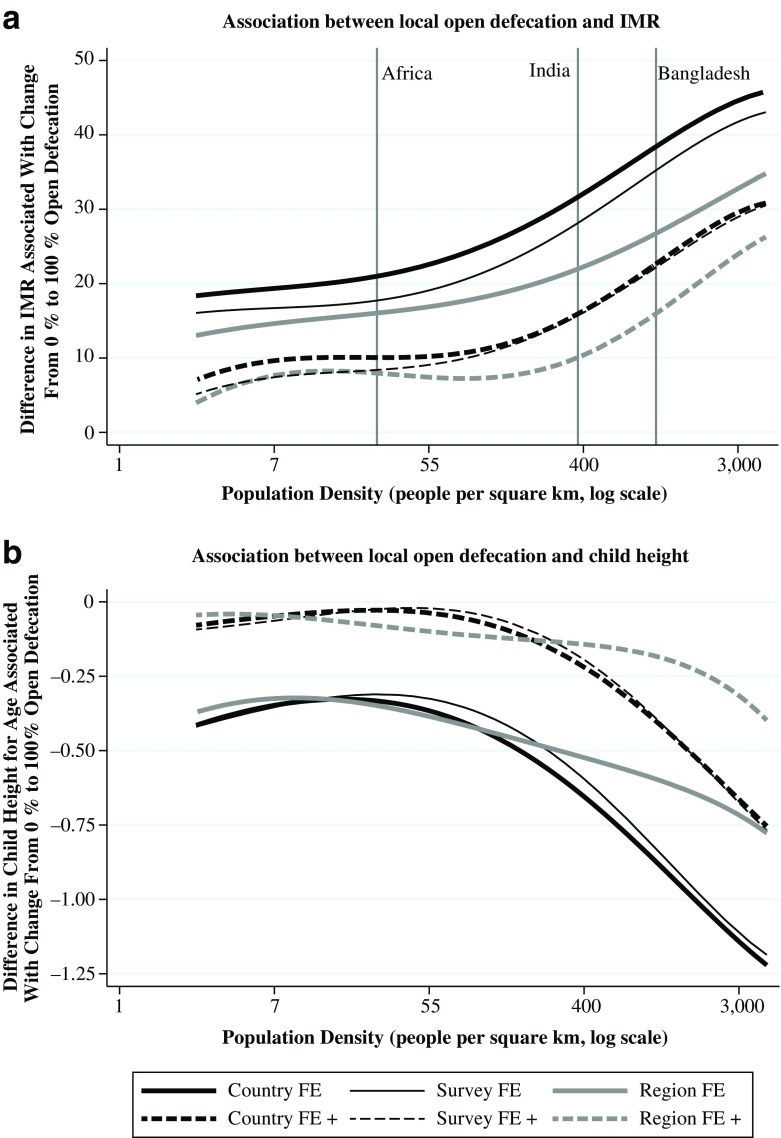
Dependence of sanitation gradient on population density, international sample

Panel A of Fig. 2 plots the dependence of the infant mortality–open-defecation gradient on population density; panel B plots the height–open-defecation gradient as a function of population density. In both cases, the same six specifications that were used in Table 3 are plotted: fixed effects at the country, survey, and region level, with and without an extended vector of controls. *F* tests with 8 degrees of freedom showing that the higher-order interaction does not improve the fit—that is, that β_2,2_ through β_2,5_ and β_3,2_ through β_3,5_ are all 0—are rejected. For example, with *F* = 8.50, *p <* .0001 in the case of country fixed effects with no controls.

Figure 2 shows that although adding controls and changing the fineness of fixed effects shifts the estimated function vertically, which changes the *level* of the sanitation-health gradient, the shape of the function—that is, the dependence of the health–open-defecation gradient on population density—remains similar. Across model specifications, the association between open defecation and infant mortality, for example, is about twice as steep in places with the average population density of Bangladesh (or in the similarly dense, largely rural Indian states of Uttar Pradesh and Bihar) as it is in places with the average population density of sub-Saharan Africa. Moreover, the function curves convexly, so the effect of population density is even greater at higher levels of population density. Because the average population density in the Bangladesh data used in the following section is especially high, these estimates predict a particularly steep sanitation-health gradient and large interaction with population density in that context.

## Population Density, Sanitation, and Child Height in Bangladesh

The preceding section shows that higher population density is robustly and uniquely associated with a steeper sanitation-health gradient. This section uses variation across time and place in local open defecation within Bangladesh (a country where open defecation has fallen sharply over recent decades) in order to provide further evidence for the internal validity of the sanitation–population density interaction.

Bangladesh is an apt case study to further interrogate the sanitation–population density interaction for two reasons. First, unlike many DHS, the Bangladesh DHS report GIS codes for PSUs. This permits us to create a more precise measure of the density of open defecation to which an individual child is exposed than we were able to use based on the international data, and also to control for fixed effects at the district level, which is a much smaller geographic area than the region that was used in the international analysis. Second, Bangladesh experienced a rapid decline in open defecation over the period we study. According to UNICEF–WHO statistics, national open defecation declined from 20.6 % in 1999 to 3.9 % in 2011 (UNICEF and WHO 2012). As a result, much of the variation that we use to identify the effect of the interaction of sanitation and population density on child height results from a reduction in the density of open defecation over time.

### Data and Summary Statistics

We combine data from the 1999, 2004, and 2011 Bangladeshi DHS, as well as from two Bangladesh censuses, to investigate the relationship among open defecation, population density, and child height. To do this, we match the PSUs of children in the DHS to political boundaries using GIS codes.

There are four levels of political disaggregation within Bangladesh. Most coarsely, Bangladesh is divided into seven divisions. Divisions are the subnational regions coded in DHS data; we refer to these as *regions* for consistency with the earlier section Population Density, Sanitation, and Child Health in Developing Countries. Regions are divided into districts, which are not reported in the DHS. With a total of 64 districts in Bangladesh, the average district has a population of approximately 2 million people. Districts are divided into subdistricts, which are then divided into unions (rural), wards (parts of cities), or *pourashava* (towns), which we abbreviate in aggregate as UWP. The average UWP had 339,906 people in the 2011 census.

Each PSU in the Bangladesh DHS is accompanied by a GIS code (publicly available on request), which includes the latitude and longitude of the PSU.^17^ We use ArcGIS 10 software and a polygon overlay technique to match PSUs from the DHS to districts and UWPs from the 2009 Local Government Engineering Department (LEGD) UWP-level map. After identifying each PSU’s UWP, we match it with a UWP-level population density from census data from Bangladesh Bureau of Statistics (2002, 2012) to create our independent variable of interest: the interaction of PSU-level open defecation with the log of UWP-level population density. The 1999 and 2004 DHS are matched to the 2001 population census of Bangladesh; the 2011 DHS is matched to the 2011 population census.^18^ Thus, each PSU is matched to a highly disaggregated measure of population density. Because the DHS are repeated, nationally representative cross-sections that do not form a panel of PSUs, it is often the case that a given UWP is not represented in more than one round of the DHS. Therefore, the smallest geographic unit for which we can include a fixed effect is the district.

#### Independent Variable of Interest

Our independent variable of interest is the interaction of the log of UWP-level population density with the fraction of households in a PSU that defecates in the open; this is the same for each child in a given survey round and PSU.

#### Dependent Variable

The dependent variable in this analysis is the height-for-age *z* score of children under 5, using the WHO 2006 reference of healthy children. For the Bangladesh analysis, we no longer use infant mortality as a dependent variable. With a sample less than 4 % as large as in the international analysis presented earlier, we are unable to precisely identify effects on infant mortality, a low probability binary outcome, using district fixed effects. Sample size is less of a constraint for continuously distributed, normalized height-for-age, which is routinely studied in samples of this size (e.g., Spears et al. 2013). Online Resource 1 presents evidence that supports an interactive effect of sanitation and population density on infant mortality. Table S2 in that supplement presents results for infant mortality that use fixed effects for region—rather than district fixed effects—and repeats falsification tests showing no similar interaction with electrification or radio ownership.

#### Summary Statistics

Table 4 reports summary statistics for the Bangladesh data set. Observations are infants and children, so averages do not generally correspond to published summary statistics representative of the population of Bangladesh. For example, if young children are disproportionately found in poorer households, our summary statistics will present a worse picture of human development. Indeed, the summary statistics reflect a poor, mainly rural population with high mortality and low maternal nutrition. However, child height, infant mortality, sanitation, maternal nutrition, and electrification all show clear improvements over the three survey rounds.

**Table 4:** Summary statistics: Bangladesh sample

	Year		
	1999	2004	2011
Height-for-Age	–1.95	–1.92	–1.62
Infant Mortality Rate	81.57	72.33	50.41
Household Open Defecation	0.199	0.141	0.128
Local Open Defecation	0.201	0.138	0.132
Population Density per km^2^	4,983	4,344	4,466
ln(Density)	7.23	7.17	7.29
Mother’s Height (cm)	150	150	151
Mother’s Age	22.72	22.59	22.43
Local Radio	0.33	0.32	0.08
Local Electricity	0.36	0.42	0.60
Urban	0.27	0.31	0.31
*n* (height-for-age)	5,435	5,978	7,743
*n* (infant mortality)	12,517	12,817	16,902

*Notes:* Observations are individual children born alive. Children are included in the summary statistics sample if they are in either the infant mortality rate sample or the height sample.

### Empirical Strategy

We identify the association between local sanitation density and child height from cross-sectional and over-time variation within districts. The GIS matching described earlier allows us to use fixed effects that are approximately 10 times finer than the seven regional fixed effects used in the international analysis. We estimate regressions with district and survey round fixed effects for children under 5 years old of the following form:

6 $$ \begin{array}{l}{height}_{idt}={\upbeta}_1 local\ {OD}_{idt}+{\upbeta}_2 \ln {(density)}_{idt}+{\upbeta}_3 local\ {OD}_{idt}\times \ln {(density)}_{idt}\\ {}+{\upbeta}_4 household\ {OD}_{idt}+{X}_{idt}\uptheta +{A}_{idt}\times {sex}_{idt}+{year}_{idt}+{\updelta}_d+{\gamma}_t+{\upvarepsilon}_{idt},\end{array} $$

where *i* indexes individual children, *d* indexes districts, and *t* indexes survey rounds. Standard errors are clustered at the district level (that is, pooling all survey rounds within a district). As with prior regressions in which height-for-age is the dependent variable, we include 120 age (in months) by sex indicators *A*_*idt*_ × *sex*_*idt*_. We also add fixed effects for the year in which a child was born, *year*_*idt*_, to account for overall time trends. As before, we control for an indicator for whether the child’s own household defecates in the open. δ_*d*_ is a district fixed effect, and γ_*t*_ is a survey round fixed effect. This strategy allows us to control for everything about a child’s district, for any potential time trends affecting height, as well as any potential survey round–specific measurement issues.

To demonstrate the robustness of our result to individual and household regression controls, we add controls, **X**_*idt*_, which are more comprehensive than those included in the international analysis, in stages:

- Birth demography: mother’s age at the child’s birth as a quadratic polynomial, indicators for multiple birth, indicators for calendar month of birth, and an indicator for being the first born to a mother;
- Household wealth: indicators for the household having electricity, a radio, a television, a bicycle, and a motorcycle or scooter;
- Maternal nutrition, anthropometry, and care: mother’s height (in centimeters), an indicator for mother’s literacy, and an indicator for breast-feeding beginning on the first day.

### Results

Table 5 presents estimates of regression Eq. (6). We find that local sanitation statistically significantly and robustly interacts with local population density to predict average child height. Adding fixed effects and controls does little to change the magnitude of the coefficient on the interaction; none of the six estimates is statistically distinguishable from the others. These coefficients suggest that a doubling of population density is approximately associated with a 0.2 height-for-age standard deviation increase in the difference in average child height between places where there is no open defecation and where there is 100 % open defecation.^19^ The stability of the coefficient on the interaction suggests that it is unlikely to be driven by an omitted variable uncorrelated with all of these controls.

**Table 5:** Open defecation interacts with population density to predict height: Bangladesh sample

	Height-for-Age *z* score					
	(1)	(2)	(3)	(4)	(5)	(6)
Local Open Defecation *×* ln(Density)	–0.372*	–0.455**	–0.332*	–0.324*	–0.261^†^	–0.278*
	(0.176)	(0.152)	(0.163)	(0.149)	(0.139)	(0.137)
Local Open Defecation	–0.654***	–0.768***	–0.624***	–0.590***	–0.364**	–0.331**
	(0.122)	(0.122)	(0.130)	(0.123)	(0.118)	(0.122)
ln(Density)	0.045^†^	0.048^†^	0.055*	0.047*	–0.007	–0.003
	(0.023)	(0.026)	(0.024)	(0.023)	(0.018)	(0.019)
Household Open Defecation	–0.227***	–0.223***	–0.214***	–0.193***	–0.079^†^	–0.034
	(0.043)	(0.042)	(0.041)	(0.041)	(0.041)	(0.040)
Mother’s Height (cm)						0.038***
						(0.004)
Age in Months *×* Sex	✓	✓	✓	✓	✓	✓
District Fixed Effects		✓	✓	✓	✓	✓
Round and Year-of-Birth Fixed Effects			✓	✓	✓	✓
Birth Demography				✓	✓	✓
Household Wealth					✓	✓
Maternal Nutrition and Care						✓
*n* (children under 5)	19,156	19,156	19,156	19,156	19,061	19,014

*Notes:* Standard errors clustered by 66 districts in parentheses. For a complete list of control variables, please see the text.

^†^ *p <* .10; *p < .05; **p < .01; ***p < .001 (two-tailed tests)

The average linear interaction in Table 5 for Bangladesh is approximately 10 times the size of the international average linear interaction in Table 3. This best linear approximation to the interaction is useful because it allows our fixed effects identification strategy and permits simple statistical significance tests with controls. However, Fig. 2 suggests that over the entire global range of variation in population density, the interaction is not linear. Instead, the dependence of the health-sanitation gradient on population density appears to be steeper at greater population densities.

By international comparison, average population density in Bangladesh is very high. Bangladeshi children, therefore, would be on the right side of panel B of Fig. 2, which predicts a particularly steep linearized interaction between population density and open defecation. Indeed, when we use the six models estimated in panel B of Fig. 2 to compute the relevant linear interaction gradients at the average population density for children the Bangladeshi sample, we find that the numerical predictions for the coefficient on the interaction range from −0.143 to −0.309. These magnitudes are larger than the global average linear interactions presented in Table 3, which range from −0.023 to −0.074, and similar to coefficients for Bangladesh found with our fixed-effects identification strategy in Table 5, which range form −0.261 to −0.455.

## Discussion and Conclusion

Our study was motivated by the observation that an interaction between sanitation and population density importantly moderates the relationship between place and early-life health outcomes. The results presented in this article sharpen our understanding of this interaction, and investigated its external and internal validity. In two separate analyses—representing two different points in a trade-off between external validity and internal validity—we find that poor sanitation is more detrimental for early-life health where population density is greater. Stated differently, population density does not have the same benefits for health where sanitation is poor. These results are biologically plausible because open defecation leads to environmental contamination with germs from feces, and these germs are more likely to cause disease where people are more likely to come in contact with them.

Although resolving long-standing debates about the health advantages or penalties of living in urban or densely populated areas is well beyond the scope of this article, our results suggest some clarifications about the importance of place for child health in developing countries. We have isolated that high population density and poor sanitation *in combination* are particularly threatening to early-life health. Our results suggest that high density *without* poor sanitation is substantially less dangerous, such that the advantages of access to health care and other resources might dominate the disadvantages of disease externalities, yielding a net health benefit of living in dense cities (Leon 2008). Additionally, urban settings with low population density may not be disadvantaged relative to rural settings with high population density.

Our result has an important implication for policymakers: for a given level of open defecation, concentrate attention on improving sanitation where population density is high, or at minimum include population density as a factor in allocation decisions. We emphasize that this result does not exclusively or even necessarily recommend that sanitation policy attend to urban places. Population density is a continuous variable, and many parts of the developing world that are classified as rural have higher population densities than places classified as urban. The latest estimates of open defecation and population density in the developing world suggest an increasing concentration of open defecation in densely populated parts of rural India, which poses a significant threat to the health of children in these regions, despite their “rural” classification.^20^

## Electronic supplementary material

ESM 1 (PDF 496 kb)

## Footnotes

1 We do not present multilevel models because, as Sastry and Hussey (2003) explained, these models require the assumption that the random effects used in the models are independent of measured covariates. This independence criterion is not met in this case; for example, more-urbanized districts have higher population density, on average.

2 For example, Root (1997) found that population density is correlated with child mortality across provinces of Zimbabwe. A study of typhoid in Dhaka showed that crowdednesss has a considerable impact on the transmission and distribution of the disease: areas with low risk of typhoid were those with the lowest population density, and those with the highest risk had the highest population density (Corner et al. 2013). Ali et al. (2002) showed that higher population density is associated with a greater risk of cholera in a rural part of Bangladesh. Grassly et al. (2006:1151) described challenges to polio eradication in densely populated Uttar Pradesh and Bihar: “[H]igh population density and poor sanitation can lead to more frequent infectious contacts and increase levels of excreted polio-virus in the environment.”

3 Brinkley (1997) and Coelho and McGuire (1997) discussed the influence of hookworm, spread by poor sanitation, on the population health and economic development of the American South. An observational study in rural Wisconsin in the United States found that a higher density of septic tanks was associated with an increased prevalence of diarrhea (Borchardt et al. 1979). Studies of the Tama River in Tokyo and the Cumberland River in Nashville, Tennessee (United States) showed that fecal bacteria concentrations, possibly originating from sewer overflows, were significantly affected by population density (Ham et al. 2009; Young and Thackston 1999). An aggregated (or “ecological”) study across three developed countries also found suggestive evidence that higher population density may be related to increased antibiotic resistance because higher interpersonal contact can lead to the spread of resistant bacteria (Bruinsma et al. 2003).

4 The relationship between the density of open defecation and child health outcomes likely depends on the kind of parasites that are present in a region and on the conditions under which they are most easily spread.

5 More information about the use of DHS data can be found in Rutstein and Rojas (2006) or online (www.dhsprogram.com).

6 DHS use two-stage random sampling. First, a PSU, which is either a rural village or a small set of urban blocks, is selected; second, households within the PSU are randomly selected.

7 Because the fraction of households in a PSU that defecates in the open is estimated from a sample, and not from data on every household in the neighborhood, this is a noisy measure of the true fraction of households in a child’s local area UNICEF defecate in the open. This random measurement error will attenuate our coefficients, so any sanitation gradient we uncover may be a lower bound. We further discuss potential consequences of measurement error in Online Resource 1 (section S.2).

8 Figure S1 in Online Resource 1 shows that our results are not sensitive to the choice of five years before the survey as a cutoff for inclusion in the sample.

9 Following standard practice using these UNICEF *z* scores, we omit any child beyond *±*6.

10 The DHS defines urban residence based on the definitions used by countries’ national statistical offices.

11 Because the variables in the regression are demeaned, we compute the difference between urban and rural, where local open defecation is 0 by $-2.4=-6.047+5.592\times \left(1 - \overline{open\  defecation}\right)$; and the difference between urban and rural where local open defecation is 100 % by $-8=-6.047-5.592\times \overline{open\  defecation}$, where $\overline{open\  defecation}=0.35$, which is the mean of $\overline{open\  defecation}$.

12 We are constrained to use variables that are available in all of the DHS.

13 We do not use a constant term with this or the other fixed effects models in the article. In such a model, the constant term is absorbed into the set of fixed effects. See Cameron and Trivedi (2010:231).

14 We obtain these estimates simply by taking a visual approximation of the coefficients on the interaction in Table 3. These are reported in the first row of panel A for infant mortality, and the first row of panel B for height-for-age.

15 Although we find little evidence that community-level measures of SES interact with population density to predict infant mortality, evidence suggests that other community-level behaviors related to the spread of infectious disease do interact with population density. Table S5 in Online Resource 1 shows the results of regressing infant mortality on interactions of community-level infectious disease behaviors, like the fraction of children with a BCG (tuberculosis) vaccine, measles vaccine, or the fraction of children sleeping under a bed net, with population density. Although some of these interactions are statistically significant, Table S5 shows that the interaction of open defecation and population density is statistically significant even after these other interactions are controlled for.

16 A high fraction of the local area being in the bottom fifth of the country-year’s asset index statistically significantly interacts with population density *without* controls, although not *with* them; although beyond the scope of this article, it is plausible that there is a special effect on health of poor people living densely close together. However, this cannot be an omitted variable in our results: our extended controls include indicators for the individual assets used to construct the asset index.

17 We drop two PSUs from the 2004 DHS where GIS information was not reported.

18 In a small number of cases, area was not available from the census, so we computed population density by dividing census population by area from LEGD data.

19 Because population density is in log, the coefficient estimate can be interpreted as indicating that a doubling of the population density is associated with a $\ln (2)\times \widehat{\upbeta}$ difference in the dependent variable. In this case, ln(2) *×* 0.3 *≈* 0.2.

20 According to the UNICEF-WHO statistics, open defecation is increasingly a South Asian, and particularly an Indian, problem. Although Bangladesh has drastically reduced open defecation, and Pakistan has seen marked improvements, there continues to be more open defecation in India than there is toilet or latrine use. More than one-half (60 %) of open defecation in the world occurs in India, a country where 70 % of households live in rural areas, and 70 % of rural households defecate in the open. Primarily rural Indian states, such as Uttar Pradesh and Bihar (home to approximately 300 million people), have population densities that exceed 800 persons per square kilometer.
