# Supplementary material for: Place and Child Health: The Interaction of Population Density and Sanitation in Developing Countries
Source: Demography. 2017 Jan 9;54(1):337–60. doi: 10.1007/s13524-016-0538-y (PMC5306240; doi:10.1007/s13524-016-0538-y)
Supplement: Supplementary file 1 — (PDF 496 kb) [file 13524_2016_538_MOESM1_ESM.pdf]

Online Resource 1

S.1 Explanations of figures and tables

Figure S1 shows that our results are not sensitive to our choice of cutoff for inclusion in the international sample used in Table 3. It plots coefficients on the interaction of population density and open defecation from column 1 of Table 3, allowing the cutoff for inclusion in the sample to range from 5 to 15 years before the survey. The point estimate is essentially numerically unchanged. The confidence intervals are wider when births further back in time are included. This is consistent with greater measurement error in the independent variable for children born more years before the survey: children who were born long before the DHS experienced somewhat different early life conditions than what is measured by the DHS. However, the stability of the main result means that there is no reason to believe that our choice of cutoff biases the estimate of the interaction of population density and open defecation.

Figure S2 shows that the distribution of the log of population densities in our international sample, used for the analysis in section 3.3, follows approximately a normal distribution.

In order to verify the appropriateness of using the log of population density in our main regression results, rather than some other transformation of the data, Figure S3 presents results of 11 estimates using Box-Cox transformations. We transform population density with Box-Cox parameter lambda at intervals of 0.1 from 0 (which is equivalent to our preferred log specification) to 1 (which is equivalent to population density being entered linearly). Because log likelihood is maximized between 0 and 0.2, and is lowest at the linear specification 1, we conclude from this figure that our log transformation is the most useful combination of model fit, theoretical appropriateness, and ease of interpretation. However, we further plot *t* statistics on the interaction for the convenience of the reader, and note that our main result is qualitatively robust to this entire range of respecifications, with the interaction statistically significant at the .10 level in all cases, including intermediate values of lambda with no principled motivation, and at the .05 level in 6 of 11 cases. Therefore, this robustness check can increase confidence that our results are not spuriously due to any fragile log modeling choice.

Table S1 lists the data sources for the population density variable used in the analysis in section 3.3.

Table S2 uses the Bangladesh data set described in section 4.1 to show results of regressions of infant mortality on the interaction of population density and open defecation. These results complement the analysis shown in 4 of the text. Column 1 shows a regression of whether or not an infant died on PSU level open defecation, population density in his/her local area, and the interaction of these two variables. Column 2 adds fixed effects for region and finds a similar, positive, interaction of open defecation and population to what was reported in column 1. Columns 3 and 4 present falsification tests that find that the interaction of community-level electrification and population density does not similarly predict IMR, nor does the interaction of community-level radio ownership and population density.

Table S3 shows the results of a mechanism check that supports our main results. In Table S3, we regress child weight-for-height (computed using the World Health Organization 2006 norms), a measure of exposure to disease in the short and medium run, on the interaction of population density and open defecation. The coefficient is negative and numerically similar across specifications; it is more precisely estimated when we control for height (column 2). Just as it is important to control for age when using height-for-age as a dependent variable (Cummins 2013), it is important to control for height when using weight-for-height as a dependent variable. This ensures that our estimation strategy is robust to any possible functional form of the mean height-weight relationship in the sample population that may differ from the WHO norms. Column 3 adds additional controls for the child's

age, sex, and their interaction.

Table S4 shows the results of a robustness check that controls for the age of the child's mother at the time of the child's birth. Column 1 of Table S4 reproduces the results of column 1 of Table 3 in the main text. Column 2 additionally controls for the mother's age at the time of the child's birth. The coefficient on the interaction of population density and open defecation is of a similar magnitude, and it is statistically significant. Column 3 reproduces the results of column 2 of Table 3, and column 4 adds a control for mother's age at the time of the child's birth. Again, the coefficient on the interaction of population density and open defecation is of a similar magnitude, and it is statistically significant.

Table S5 shows the results of a robustness check that adds interactions of population density and community-level infectious disease behaviors other than open defecation to regressions of the form presented in column 1 of Table 3 in the main text. This table shows that community-level infectious disease behaviors, like the fraction of children with a BCG vaccine (column 2), or a measles vaccine (column 3), do interact with population density to predict infant mortality. This is not surprising: we would expect infectious diseases like BCG and measles to spread more easily in places where unvaccinated children live closer together than they do in places where children live farther apart. There is also suggestive, though not statistically significant, evidence that whether or not children under 5 sleep under a bed net (column 4) may also interact with population density to predict infant mortality. However, our main result, that the fraction of the PSU practicing open defecation interacts with population density to predict infant mortality, is nevertheless robust to controlling for the interaction of each of these measures of community-level infectious disease behavior with population density. The interaction of population density and open defecation is not merely proxying for the interaction of population density and other community-level infectious disease behaviors.

S.2 Measurement error in the independent variables

This section of the appendix considers the implications of measurement error for our finding that population density interacts with local open defecation to predict child health outcomes. One source of measurement error is that our data on open defecation come from a sample survey, rather than a census, so local open defecation to which a child is exposed is measured with error. Because the DHS chose a random sample of households in each PSU, this source of measurement error is classical measurement error. Classical measurement error leads to attenuation bias; that is, it biases coefficients toward zero. Therefore, measurement error in the open defecation variable would bias *against* finding a result.

The population density data present a second source of measurement error. We would like to be able to measure population density in a child's local area in the year of her birth, but our data on population density in the international sample are at the region level, and are from a single year, rather than the year of the child's birth. We do not think that using population density data from a year other than that of the child's birth is biasing in favor of our results because we see no systematic change in our results if births further back before the survey are included in the sample, other than increasing noise in the coefficient estimate (see Figure S1). This is consistent with increasing measurement error, but inconsistent with bias. Nor do we believe that using data from the region, rather than the local level, in our international sample, would bias in favor of our result. Some evidence for this is that we find quantitatively similar robust estimates in Bangladesh as we do in our international sample; in the Bangladeshi case our population densities are measured with considerably less error because the geographic units are much smaller than DHS sub-national regions in our international analysis.

Finally, because our coefficient of interest is on an interaction, it is also useful to ask: what is the effect of measurement error in an independent variable on the estimated coefficient of an interaction?

Jaccard and Wan (1995) have shown that the familiar attenuation bias effect of measurement error is extended to the case of interactions: we would expect the coefficient on the interaction to be attenuated towards zero.

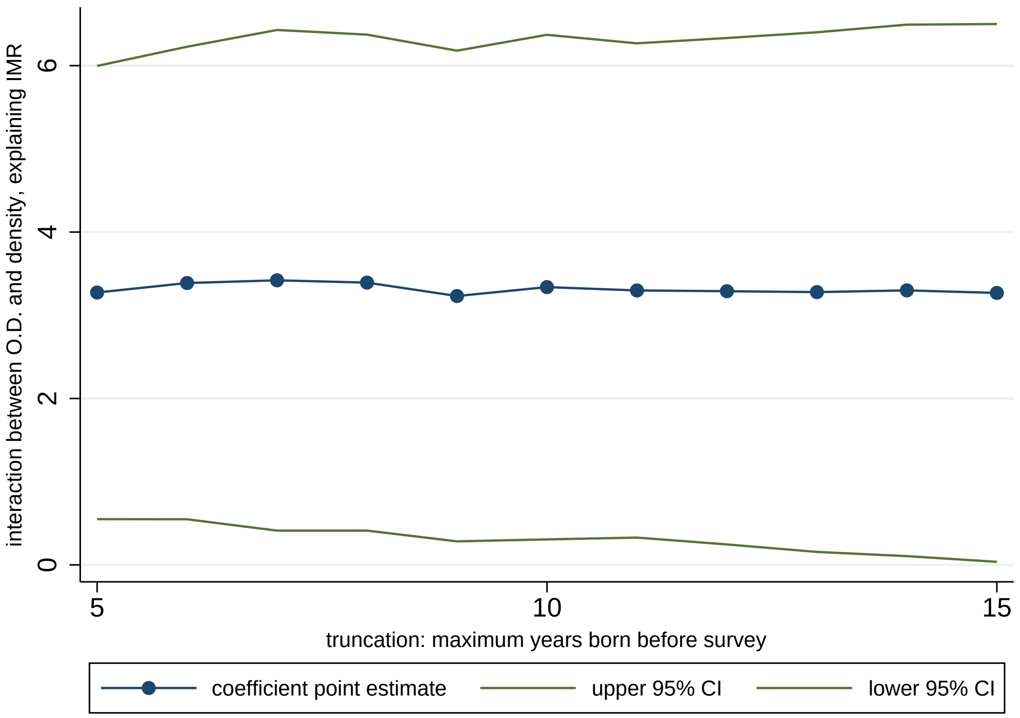

Figure S1: Coefficient on the population density-open defecation interaction does not depend on sample truncation

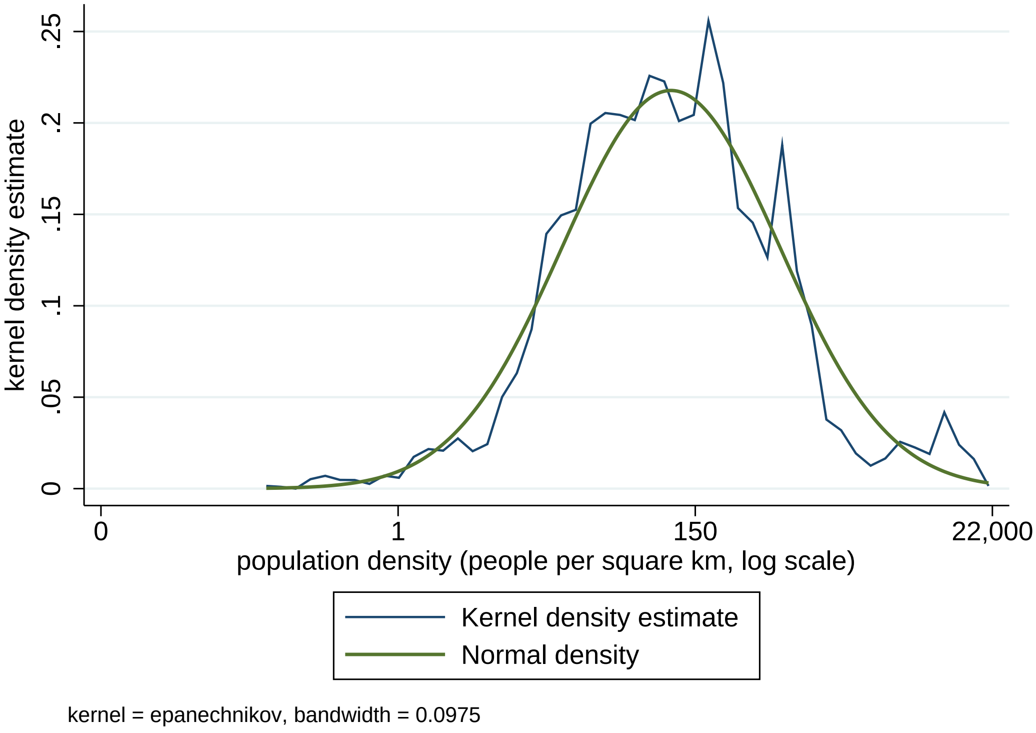

Figure S2: Distribution of population densities, international sample

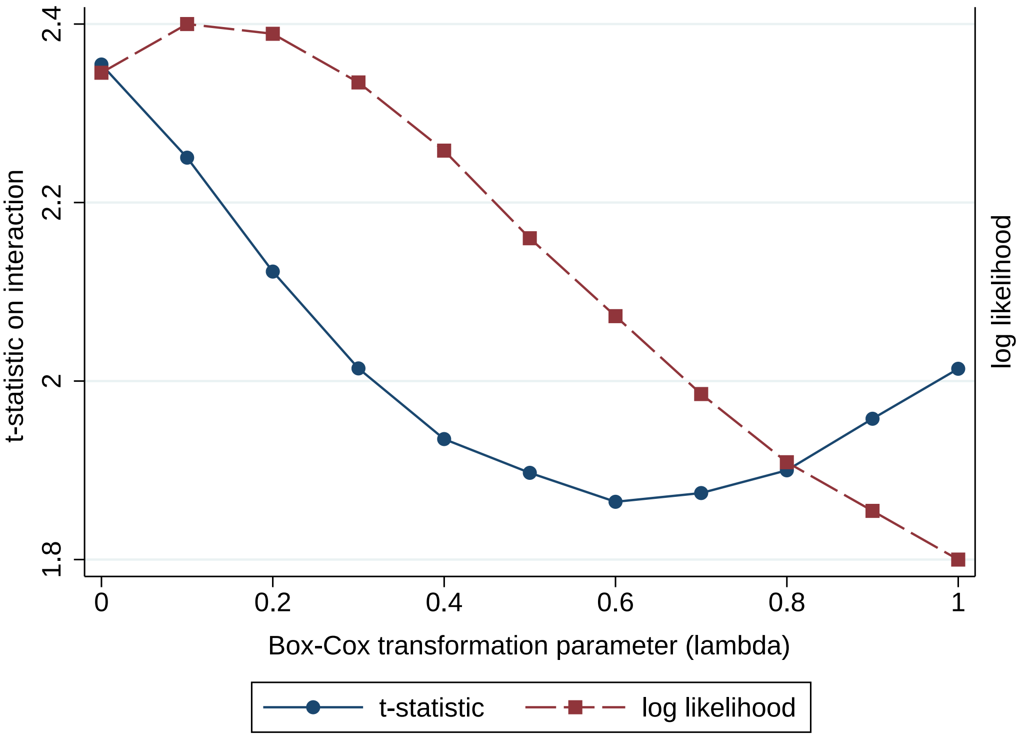

Figure S3: Box-cox transformation parameters and associated *t*-statistics for regression in column 1 of table 3

Table S1: International sample of 172 DHS

country	year	online source	ultimate source
Albania	2008	wikipedia	Institute of Statistics of Albania. 2011.
Armenia	2000	geohive	National Statistical Service. (2001 & 2011)
Armenia	2005	geohive	National Statistical Service. (2001 & 2011)
Armenia	2010	geohive	National Statistical Service. (2001 & 2011)
Azerbaijan	2006	geohive	State Statistical Committee.
Bangladesh	1993	wikipedia	Bureau of Statistics, Population Census Wing.
Bangladesh	1996	wikipedia	Bureau of Statistics, Population Census Wing.
Bangladesh	1999	wikipedia	Bureau of Statistics, Population Census Wing.
Bangladesh	2004	wikipedia	Bureau of Statistics, Population Census Wing.
Bangladesh	2007	wikipedia	Bureau of Statistics, Population Census Wing.
Bangladesh	2011	wikipedia	Bureau of Statistics, Population Census Wing.
Benin	1996	statoids	Troisième Recensement General de la Population et de l'Habitation.
Benin	2001	statoids	Troisième Recensement General de la Population et de l'Habitation.
Benin	2006	statoids	Troisième Recensement General de la Population et de l'Habitation.
Bolivia	1998	statoids	Instituto Nacional de Estadística, Table of department populations.
Bolivia	2003	statoids	Instituto Nacional de Estadística, Table of department populations.
Brazil	1991	geohive	IBGE , Brazil.
Brazil	1996	geohive	IBGE , Brazil.
Burkina Faso	1993	wikipedia	National Census. (2006)
Burkina Faso	1998	wikipedia	National Census. (2006)
Burkina Faso	2003	geohive	Institut National de la Statistique et de la Demographie.
Burkina Faso	2010	geohive	Institut National de la Statistique et de la Demographie.
Burundi	2010	geohive	ISTEEBU, Bujumbura, Burundi.
Cambodia	2000	geohive	National Institute of Statistics, Cambodia.
Cambodia	2005	geohive	National Institute of Statistics, Cambodia.
Cambodia	2010	geohive	National Institute of Statistics, Cambodia.
Cameroon	1991	geohive	National Institute of Statistics, Cameroon.
Cameroon	1998	geohive	National Institute of Statistics, Cameroon.
Cameroon	2004	geohive	National Institute of Statistics, Cameroon.
Cameroon	2011	geohive	National Institute of Statistics, Cameroon.
Central African Republic	1994	geohive	Census 2003, Central African Republic.
Chad	1996	statoids	Census of Chad. (1993)
Chad	2004	statoids	Census of Chad. (2009)
Colombia	1990	geohive	Departamento Administrativo Nacional de Estadística.
Colombia	1995	geohive	Departamento Administrativo Nacional de Estadística.

Continued on next page

Table S1: International sample of 172 DHS

country	year	online source	ultimate source
Colombia	2000	wikipedia	Census of Colombia. (2005)
Colombia	2005	wikipedia	Census of Colombia. (2005)
Colombia	2010	wikipedia	Census of Colombia. (2005)
Comoros	1996	geohive	Commissariat General du Plan, Union des Comores.
Congo	2007	geohive	http://www.cd.undp.org
Congo Brazzaville	2005	geohive	Centre National de la Statistique et des Études Économiques.
Congo Brazzaville	2011	geohive	Centre National de la Statistique et des Études Économiques.
Côte d'Ivoire	1994	citypopulation.de	Institut National de la Statistique.
Côte d'Ivoire	1998	citypopulation.de	Institut National de la Statistique.
Côte d'Ivoire	2011	citypopulation.de	Institut National de la Statistique.
Dominican Republic	1991	geohive	Oficina Nacional de Estadística.
Dominican Republic	1996	geohive	Oficina Nacional de Estadística.
Dominican Republic	1999	geohive	Oficina Nacional de Estadística.
Dominican Republic	2002	geohive	Oficina Nacional de Estadística.
Dominican Republic	2007	geohive	Oficina Nacional de Estadística.
Egypt	1992	statoids	Central Agency for Public Mobilization and Statistics.
Egypt	1995	statoids	Central Agency for Public Mobilization and Statistics.
Egypt	2000	statoids	Central Agency for Public Mobilization and Statistics.
Egypt	2005	statoids	Central Agency for Public Mobilization and Statistics.
Egypt	2008	statoids	Central Agency for Public Mobilization and Statistics.
Ethiopia	2000	geohive	CSA, Ethiopia.
Ethiopia	2005	geohive	CSA, Ethiopia.
Ethiopia	2011	geohive	CSA, Ethiopia.
Gabon	2000	geohive	Direction Generale de la Statistique et des Etudes Economiques.
Gabon	2012	geohive	Direction Generale de la Statistique et des Etudes Economiques.
Ghana	1993	statsghana	Ghana Statistical Service.
Ghana	1998	statsghana	Ghana Statistical Service.
Ghana	2003	statsghana	Ghana Statistical Service.
Ghana	2008	statsghana	Ghana Statistical Service.
Guatemala	1995	indexmundi	FAO and World Bank population estimates.
Guinea	1999	geohive	Institut National de la Statistique, Guinea.
Guinea	2005	geohive	Institut National de la Statistique, Guinea.
Guyana	2009	geohive	Statistics Guyana.
Haiti	1994	geohive	Institut Haïtien de Statistique et d'Informatique (IHSI), Haiti.
Haiti	2000	geohive	Institut Haïtien de Statistique et d'Informatique (IHSI), Haiti.
Haiti	2005	geohive	Institut Haïtien de Statistique et d'Informatique (IHSI), Haiti.
Haiti	2012	geohive	Institut Haïtien de Statistique et d'Informatique (IHSI), Haiti.

Continued on next page

Table S1: International sample of 172 DHS

country	year	online source	ultimate source
Honduras	2005	geohive	Instituto Nacional de Estadística, Honduras.
Honduras	2011	geohive	Instituto Nacional de Estadística, Honduras.
India	1992	censusindia.gov	Census of India. (1991)
India	1998	censusindia.gov	Census of India. (2001)
India	2005	wikipedia	Census of India. (2011)
Indonesia	2002	geohive	Biro Pusat Statistik.
Indonesia	2007	geohive	Biro Pusat Statistik.
Indonesia	2012	geohive	Biro Pusat Statistik.
Jordan	1997	geohive	Department of Statistics, Amman, Jordan.
Jordan	2002	indexmundi	FAO and World Bank population estimates.
Kazakhstan	1995	geohive	National Statistical Agency of Kazakhstan.
Kazakhstan	1999	geohive	National Statistical Agency of Kazakhstan.
Kenya	1993	statoids	Census of Kenya. (1999)
Kenya	1998	statoids	Census of Kenya. (1999)
Kenya	2003	geohive	Kenya National Bureau of Statistics.
Kenya	2008	geohive	Kenya National Bureau of Statistics.
Kyrgyz Republic	1995	geohive	National Statistical Committee, Kyrgyz Republic.
Lesotho	2004	geohive	Lesotho Bureau of Statistics.
Lesotho	2009	geohive	Lesotho Bureau of Statistics.
Liberia	2007	wikipedia	2008 National Population and Housing Census.
Madagascar	1992	statoids	Census of Madagascar. (1993)
Madagascar	1997	statoids	Census of Madagascar. (1993)
Madagascar	2003	statoids	Census of Madagascar. (1993)
Madagascar	2008	geohive	Institut National de la Statistique, Madagascar.
Malawi	1992	geohive	National Statistical Office, Malawi.
Malawi	2000	geohive	National Statistical Office, Malawi.
Malawi	2004	geohive	National Statistical Office, Malawi.
Malawi	2010	geohive	National Statistical Office, Malawi.
Maldives	2009	wikipedia	Census of Maldives. (2006)
Mali	1995	geohive	Institut National de la Statistique du Mali, Mali.
Mali	2001	geohive	Institut National de la Statistique du Mali, Mali.
Mali	2006	geohive	Institut National de la Statistique du Mali, Mali.
Moldova	2005	geohive	Department of Statistics and Sociological Analysis, Moldova.
Morocco	1992	statoids	Europa World Year Book. (2001)
Morocco	2003	geohive	Haut Commissariat au Plan, Morocco.
Mozambique	1997	geohive	INE, Mozambique.
Mozambique	2003	geohive	INE, Mozambique.
Mozambique	2011	geohive	INE, Mozambique.
Namibia	1992	indexmundi	FAO and World Bank population estimates.
Namibia	2000	geohive	Namibia Statistics Agency.
Namibia	2006	geohive	Namibia Statistics Agency.
Nepal	1996	geohive	Central Bureau of Statistics, Kathmandu, Nepal.
Nepal	2001	geohive	Central Bureau of Statistics, Kathmandu, Nepal.
Nepal	2006	geohive	Central Bureau of Statistics, Kathmandu, Nepal.

Continued on next page

Table S1: International sample of 172 DHS

country	year	online source	ultimate source
Nepal	2011	wikipedia	National Population and Housing Census. (2011)
Nicaragua	1998	geohive	INIDE, Nicaragua.
Nicaragua	2001	geohive	INIDE, Nicaragua.
Niger	1992	geohive	Institut National de la Statistique, Niger.
Niger	1998	geohive	Institut National de la Statistique, Niger.
Niger	2006	geohive	Institut National de la Statistique, Niger.
Nigeria	1999	indexmundi	FAO and World Bank population estimates.
Nigeria	2003	indexmundi	FAO and World Bank population estimates.
Nigeria	2008	indexmundi	FAO and World Bank population estimates.
Pakistan	1990	geohive	Pakistan Census Organisation, Pakistan.
Pakistan	2006	geohive	Pakistan Census Organisation, Pakistan.
Peru	1991	geohive	INEI, Peru.
Peru	1996	geohive	INEI, Peru.
Peru	2000	geohive	INEI, Peru.
Philippines	1993	statoids	Census 2000 of Philippines.
Philippines	1998	geohive	National Statistics Office, Philippines.
Philippines	2003	geohive	National Statistics Office, Philippines.
Philippines	2008	geohive	National Statistics Office, Philippines.
Rwanda	1992	geohive	National Institute of Statistics of Rwanda (NISR).
Rwanda	2000	statoids	Census of Rwanda. (2002)
Rwanda	2005	statoids	Census of Rwanda. (2002)
Rwanda	2010	geohive	National Institute of Statistics of Rwanda (NISR).
São Tomé and Príncipe	2008	geohive	Instituto Nacional de Estatística, São Tomé and Príncipe.
Senegal	1992	indexmundi	FAO and World Bank population estimates.
Senegal	1997	indexmundi	FAO and World Bank population estimates.
Senegal	2005	geohive	ANSD, Senegal.
Senegal	2010	geohive	ANSD, Senegal.
Sierra Leone	2008	geohive	Statistics Sierra Leone, Sierra Leone.
South Africa	1998	geohive	Statistics South Africa & The Local Government Handbook.
Swaziland	2006	geohive	CSO, Swaziland and the National Development Data Centre.
Tanzania	1991	indexmundi	FAO and World Bank population estimates.
Tanzania	1996	geohive	National Bureau of Statistics, Tanzania.
Tanzania	1999	geohive	National Bureau of Statistics, Tanzania.
Tanzania	2004	geohive	National Bureau of Statistics, Tanzania.
Tanzania	2010	geohive	National Bureau of Statistics, Tanzania.
Timor-Leste	2009	geohive	Direcção Nacional de Estatística, Timor-Leste.
Togo	1998	wikipedia	Direction Générale de la Statistique et de la Comptabilité Nationale.
Turkey	1993	indexmundi	FAO and World Bank population estimates.
Turkey	1998	indexmundi	FAO and World Bank population estimates.
Turkey	2003	indexmundi	FAO and World Bank population estimates.
Uganda	1995	geohive	Uganda Bureau of Statistics.

Continued on next page

Table S1: International sample of 172 DHS

country	year	online source	ultimate source
Uganda	2000	geohive	Uganda Bureau of Statistics.
Uganda	2006	geohive	Uganda Bureau of Statistics.
Uganda	2011	geohive	Uganda Bureau of Statistics.
Ukraine	2007	indexmundi	FAO and World Bank population estimates.
Uzbekistan	1996	indexmundi	FAO and World Bank population estimates.
Vietnam	1997	geohive	General Statistical Office, Vietnam.
Vietnam	2002	geohive	General Statistical Office, Vietnam.
Yemen	1991	indexmundi	FAO and World Bank population estimates.
Zambia	1992	geohive	Central Statistical Office, Zambia.
Zambia	1996	geohive	Central Statistical Office, Zambia.
Zambia	2001	geohive	Central Statistical Office, Zambia.
Zambia	2007	geohive	Central Statistical Office, Zambia.
Zimbabwe	1994	geohive	Central Statistical Office, Zimbabwe.
Zimbabwe	1999	geohive	Central Statistical Office, Zimbabwe.
Zimbabwe	2005	geohive	Central Statistical Office, Zimbabwe.

Table S2: Open defecation interacts with population density to predict infant mortality, Bangladesh

	(1)	(2)	(3)	(4)
	dependent variable: IMR (deaths per 1,000)			
local open defecation	21.92†	26.76*		
× ln(density)	(12.56)	(12.35)		
local open defecation	30.93**	25.73*		
	(10.82)	(12.54)		
local electrification			-1.08	
× ln(density)			(4.57)	
local electrification			-35.87***	
			(5.18)	
local radio ownership				3.64
× ln(density)				(5.98)
local radio ownership				27.80***
				(8.63)
ln(density)	-0.635	-0.006	3.68†	-3.15**
	(1.70)	(1.68)	(2.14)	(1.19)
household open defecation		9.91†	7.15	13.51**
		(5.15)	(4.48)	(4.39)
girl		-9.21***	-9.18***	-9.25***
		(2.47)	(2.47)	(2.48)
elapsed months, birth to survey		0.271***	0.275***	0.274***
		(0.041)	(0.041)	(0.041)
region fixed effects		$F = 9.58$	$F = 10.72$	$F = 10.07$
		$p = 0.0000$	$p = 0.0000$	$p = 0.0000$
n (live births)	41,852	41,852	41,852	41,852

Standard errors clustered by survey PSUs in parentheses. Two-sided p-values: † $p < 0.10$, * $p < 0.05$, ** $p < 0.01$ *** $p < 0.001$.

Table S3: Density of open defecation predicts child weight-for-height, international sample

	(1)	(2)	(3)
	weight-for-height		
local open defecation			
X *ln*(density)	-0.0359	-0.0385+	-0.0496*
	(0.0219)	(0.0218)	(0.0219)
local open defecation	-0.0485	-0.0468	-0.0416
	(0.0937)	(0.0939)	(0.0925)
ln(density)	0.0186*	0.0196*	0.0261**
	(0.00759)	(0.00762)	(0.00824)
household OD	-0.0683***	-0.0687***	-0.0898***
	(0.0161)	(0.0158)	(0.0177)
height (in cm)		-0.00492**	-0.0363***
		(0.00163)	(0.00674)
female			0.0444**
			(0.0139)
female X age-in-months			-0.00188***
			(0.000426)
Age-in-months			0.0285***
			(0.00503)
country Fes	✓	✓	✓
n	292011	292011	292011

Standard errors clustered by survey PSUs in parentheses. Two-sided p-values: † $p < 0.10$, * $p < 0.05$, ** $p < 0.01$ *** $p < 0.001$.

Table S4: Results are not sensitive to controlling for mother's age at child's birth, international sample

	(1)	(2)	(3)	(4)
	IMR			
local open defecation				
X *ln*(density)	3.273*	3.204*	2.271*	2.255*
	(1.390)	(1.360)	(1.049)	(1.041)
local open defecation	26.37***	26.39***	12.69***	12.57***
	(2.347)	(2.325)	(2.245)	(2.223)
ln(density)	-0.333	-0.344	0.516	0.528
	(0.645)	(0.634)	(0.518)	(0.518)
household OD	6.246***	6.281***	3.102**	3.068**
	(1.711)	(1.717)	(1.049)	(1.040)
mom's age at birth		-0.473***		-0.400*
		(0.109)		(0.182)
extended controls			✓	✓
country FEs	✓	✓	✓	✓
n	1109116	1109116	942350	942350

Standard errors clustered by survey PSUs in parentheses. Two-sided p-values: † $p < 0.10$, * $p < 0.05$, ** $p < 0.01$ *** $p < 0.001$.

Table S5: Results are robust to controlling for interaction of population density and other community-level infectious disease behaviors

	(1)	(2)	(3)	(4)
local open defecation	3.244*	1.979*	2.180*	3.184+
× *ln*(density)	(1.372)	(0.905)	(1.024)	(1.613)
local BCG vaccine		-4.932**		
× *ln*(density)		(1.540)		
local measles vaccine			-2.448+	
× *ln*(density)			(1.310)	
local bed net use				-2.523
× *ln*(density)				(2.048)
local open defecation	11.625+	6.326	4.664	0.258
	(6.218)	(4.587)	(5.142)	(6.352)
local BCG vaccine		-28.230***		
		(7.613)		
local measles vaccine			-47.606***	
			(6.819)	
local bed net use[a]				-5.982
				(9.016)
ln(density)	-1.468**	3.688*	1.004	-0.464
	(0.443)	(1.393)	(1.038)	(1.075)
household OD	6.165***	5.960***	5.887***	5.126***
	(1.739)	(1.664)	(1.634)	(1.167)
country fixed effect	✓	✓	✓	✓
n (live births)	1087033	1080212	1080207	375236

Standard errors clustered by survey PSUs in parentheses. Two-sided p-values: † $p < 0.10$, * $p < 0.05$, ** $p < 0.01$ *** $p < 0.001$. [a]Fewer observations are included in column 4 because only certain African countries collected data on whether children under five slept under a bed net the night before the survey.
